# Risk factors for chronic postsurgical pain following minimally invasive thoracic surgery

**DOI:** 10.3389/fsurg.2025.1742042

**Published:** 2026-01-12

**Authors:** Li Yongjun, Wu Weinian

**Affiliations:** Xinjiang 474 Hospital, Urumqi, Xinjiang Uygur Autonomous Region, China

**Keywords:** chronic postsurgical pain, intercostal suture, logistic regression, minimally invasive thoracic surgery, preoperative anxiety, risk factors, scar length, shoulder abduction pain

## Abstract

**Objective:**

This study aimed to systematically identify independent risk factors for chronic postsurgical pain (CPSP) in patients undergoing minimally invasive pulmonary surgery, thereby providing an evidence-based foundation for the early identification of high-risk patients and the development of targeted preventive strategies.

**Methods:**

A case-control study design was employed. A total of 280 patients who underwent minimally invasive thoracic surgery between January 2022 and June 2024 were enrolled and categorized into a CPSP group (*n* = 48) and a non-CPSP group (*n* = 232) based on the presence of CPSP at 3 months postoperatively. Baseline characteristics, surgical features, and perioperative pain indicators—including visual analog scale (VAS) scores for pain at rest, during coughing, and during shoulder abduction assessed daily from postoperative day 1 to day 6—were prospectively collected. The occurrence of CPSP was evaluated at the 3-month follow-up. Univariate and multivariate logistic regression analyses were used to screen for independent factors influencing CPSP, and the predictive performance of these factors was assessed using receiver operating characteristic (ROC) curve analysis.

**Results:**

Univariate analysis revealed that preoperative anxiety, preoperative pain, surgical approach, intercostal suture, scar length, and postoperative shoulder abduction pain were significantly associated with CPSP development (*P* < 0.05). Multivariate logistic regression analysis ultimately identified postoperative shoulder abduction pain (OR = 1.893, 95% CI: 1.432–2.502, *P* < 0.001), scar length (OR = 1.240, 95% CI: 1.049–1.466, *P* = 0.011), and preoperative anxiety (OR = 3.089, 95% CI: 1.201–7.943, *P* = 0.019) as independent risk factors for CPSP, while intercostal suture (OR = 0.234, 95% CI: 0.074–0.736, *P* = 0.013) was an independent protective factor. Predictive performance analysis showed that postoperative shoulder abduction pain had the best predictive value [Area Under the Curve (AUC) = 0.821], with an optimal cut-off value of >3.5 points.

**Conclusion:**

For patients undergoing minimally invasive thoracic surgery, higher early postoperative (within 6 days) shoulder abduction pain scores, greater scar length, and the presence of preoperative anxiety are significant independent risk factors for developing CPSP at 3 months, whereas the use of the intercostal suture technique demonstrates a protective effect. Clinical practice should emphasize enhanced monitoring and management of postoperative shoulder abduction pain, the proactive adoption of protective surgical techniques, and attention to patients’ preoperative psychological state to effectively reduce the risk of CPSP.

## 1 Introduction

Chronic postsurgical pain (CPSP) is defined as pain that persists beyond the normal healing period, typically lasting for at least 3 months after a surgical procedure. Its global incidence ranges approximately from 10% to 50% (1, 2). Among various types of surgery, thoracic procedures carry a particularly high risk for CPSP development, with reported incidence rates reaching up to 57% (3). This elevated risk is largely attributable to the specific incision sites involved and the susceptibility of the intercostal nerves to traction or injury (4). CPSP not only significantly impairs patients’ quality of life and physical function but can also lead to psychological issues such as anxiety and depression, imposing a substantial long-term healthcare burden. With the continuously rising volume of surgical procedures worldwide, the prevention and management of CPSP have become a major clinical challenge (5).

There is a general consensus within the academic community that proactive measures taken during the perioperative period to prevent the onset of CPSP represent a more effective management strategy compared to treating established pain (6). The cornerstone of effective prevention lies in the systematic identification and confirmation of independent risk factors closely associated with CPSP development (7). Although numerous studies suggest that age, sex, history of preoperative pain, psychological factors, and surgical techniques may influence the risk of CPSP, substantial evidence indicates that the intensity of acute perioperative pain and the quality of its management are among the most direct and powerful predictors for CPSP (8–10).

This study focuses on patients undergoing minimally invasive pulmonary surgery. We systematically collected multiple clinical indicators related to acute pain during the perioperative period. Employing a case-control design and utilizing both univariate and multivariate logistic regression analyses, this study aims to identify independent risk factors associated with the occurrence of CPSP from a broad set of potential variables. The ultimate goal is to provide an evidence-based foundation for the early identification of high-risk patients and the formulation of targeted interventions, thereby contributing to the improvement of long-term recovery quality for patients undergoing thoracic surgery.

## 2 Materials and methods

### 2.1 General information

This case-control study was conducted with the approval of the Ethics Committee of Xinjiang 474 Hospital. Patients who underwent minimally invasive thoracic surgery between January 2022 and June 2024 were screened from the postoperative pain follow-up database established by our hospital's Department of Thoracic Surgery. A total of 280 patients were ultimately enrolled in the study. Based on the presence of Chronic Postsurgical Pain (CPSP) at the 3-month postoperative follow-up, they were categorized into two groups: the CPSP group (*n* = 48) and the non-CPSP group (*n* = 232).

The inclusion criteria were as follows: (1) age between 18 and 75 years; (2) body mass index (BMI) ranging from 18.5 to 30.0 kg/m^2^; (3) American Society of Anesthesiologists (ASA) physical status classification of I to III; (4) scheduled for elective uniportal or multiportal video-assisted thoracoscopic pulmonary surgery.

The exclusion criteria were: (1) a history of chronic pain in the chest or shoulder (duration ≥3 months) preoperatively, or conditions such as frozen shoulder or thoracic deformities that could interfere with the assessment of shoulder movement-related pain postoperatively; (2) a history of psychiatric disorders (e.g., depression, anxiety disorders requiring medication) or cognitive impairment; (3) inability to cooperate with pain scoring and postoperative follow-up; (4) long-term (≥6 months preoperatively) regular use of medications that could affect pain perception, such as opioid analgesics, antidepressants, or anticonvulsants; (5) concomitant severe neuropathy, autoimmune diseases, or end-stage organ failure; (6) conversion to thoracotomy during surgery or concurrent major surgery at other sites; (7) missing critical clinical data.

### 2.2 Anesthetic program

A standardized general anesthesia protocol was used for all patients. Anesthesia was induced intravenously with etomidate, sufentanil, and cisatracurium. A double-lumen endotracheal tube was inserted under video laryngoscopy, and its position was confirmed by fiberoptic bronchoscopy. Anesthesia was maintained with continuous intravenous infusions of propofol and remifentanil to maintain a Bispectral Index (BIS) between 40 and 60. One-lung ventilation was initiated as required during surgery. At the end of surgery, the anesthetic infusions were discontinued. The patient was transferred to the Post-Anesthesia Care Unit (PACU), where the tracheal tube was removed after neuromuscular blockade reversal, and the patient was discharged to the ward upon meeting standard criteria. The detailed drug dosages, ventilation parameters, and hemodynamic management protocol are provided in Supplementary Material 1.

### 2.3 Surgical technique

All surgeries were performed by experienced thoracic surgeons under general anesthesia. Two primary minimally invasive approaches were included in this study: the axillary approach and the posterolateral approach. Surgery was conducted using standard multi-port or uniport video-assisted thoracoscopic techniques. Key procedural steps included: making an incision in the selected intercostal space with the use of a wound protector; consciously avoiding unnecessary clamping, electrocautery, or traction on the intercostal neurovascular bundle; and using endoscopic staplers for vascular and bronchial dissection following lung parenchymal resection (lobectomy, segmentectomy, or wedge resection). Regarding wound closure, the use of the intercostal suture technique was specifically documented. A chest tube was placed through a separate small incision. All procedures adhered to the same principles of multimodal analgesic management.

### 2.4 Data collection and outcomes

This study employed a combination of prospective data collection and retrospective medical record review to systematically acquire the required variables. Data sources included the hospital's electronic medical record (EMR) system, the anesthesia clinical information system, operating room records, and a specifically designed standardized postoperative follow-up database.

A rigorous data quality control process was implemented. All data were independently collected by two research assistants who were blinded to the study group allocation, using pre-designed electronic data collection forms. Following data collection, a cross-checking procedure was performed. Any discrepancies identified were adjudicated by a third senior researcher through a review of the original medical records.

(1) Baseline Characteristics and Preoperative Assessment

Demographic characteristics, including age, sex, height, and weight, were collected for all patients, and the Body Mass Index (BMI) was calculated. The patients’ overall physical status was assessed using the American Society of Anesthesiologists (ASA) classification system. Comorbidities, such as diabetes mellitus, hypertension, and coronary heart disease, were systematically documented. Preoperative pain status was ascertained via structured interviews, strictly adhering to the ICD-11 criteria (11, 12) to record the presence of chronic pain persisting or recurring for more than 3 months.

Furthermore, preoperative anxiety was screened by uniformly trained nurses asking the direct question, “Do you often feel nervous or anxious?”; the response was recorded as “Yes” or “No”.

(2) Intraoperative and Perioperative Management Parameters
  (i) Surgical characteristics: The surgical approach, specific procedure type, duration of surgery, and duration of anesthesia were meticulously recorded.
  (ii) Surgical technique details: The use of the intercostal suture technique was documented. This technique refers to suturing the internal intercostal muscle and parietal pleura layers precisely when approximating the intercostal space, consciously avoiding incorporating and compressing the intercostal neurovascular bundle (which runs between the internal and innermost intercostal muscles) through blunt dissection or the use of small-gauge instruments. Whether the latissimus dorsi muscle was preserved was also documented.
  (iii) Incision and drainage: Scar length was measured and recorded by the attending physician on postoperative day 3 using a precise graduated scale. The number of indwelling drainage tubes was also recorded.
  (iv) Anesthesia and analgesia management: The primary regional analgesia technique used postoperatively was documented.
  (v) Regional anesthesia: All patients received a preoperatively placed, ultrasound-guided regional nerve block as a core component of multimodal analgesia. The standardized technique was either a Thoracic Paravertebral Block (TPVB) or a Serratus Anterior Plane Block (SAPB), covering the surgical incision level and one intercostal space above and below. The block was performed using 0.375% ropivacaine with a total volume of 20–30 mL. Successful blockade was confirmed by preoperative sensory testing.
  (vi) Postoperative analgesia management: All patients adhered to a unified multimodal analgesia protocol. Baseline analgesia included: acetaminophen (1 g IV every 8 h) and a selective COX-2 inhibitor (e.g., parecoxib 40 mg IV every 12 h for 48 h). Patient-controlled intravenous analgesia (PCIA) with sufentanil (basal infusion 0.5–1 μg/h, bolus dose 2–3 μg, lockout interval 8–10 min) was available as rescue analgesia. The Acute Pain Service team assessed analgesic efficacy and side effects daily, adjusting PCIA parameters or adding adjuvants (e.g., gabapentin) per protocol. The total opioid consumption (in sufentanil equivalents) during the first 72 postoperative hours was recorded.
(3) Postoperative Pain Assessment and Follow-up
  (i) Acute pain monitoring (13): From postoperative day 1 to day 6, dedicated pain nurses assessed pain intensity daily between 8:00 and 10:00 AM using the Visual Analog Scale (VAS) for the following three states:
  (ii) Pain at rest (14): The pain level while the patient was lying quietly in a supine position.
  (iii) Pain during coughing: The maximum pain intensity experienced during a single deep cough.
  (iv) Pain during shoulder abduction (15): The maximum VAS score recorded while the patient slowly abducted the arm on the surgical side to 90° in the coronal plane. For use in the logistic regression model, the maximum VAS score of shoulder abduction pain assessed daily from postoperative day 1 to day 6 was calculated and included in the analysis as a continuous variable.
  (v) Pain trajectory analysis (16): Based on the dynamic shoulder pain scores from postoperative days 1–5, different pain evolution patterns were identified using Group-Based Trajectory Modeling (GBTM).
  (vi) Chronic pain assessment: A standardized evaluation was conducted at 3 months postoperatively via outpatient review or telephone follow-up. Chronic Postsurgical Pain (CPSP) was strictly defined according to ICD-11 classification criteria as pain that develops or significantly intensifies in a surgical area, persisting for at least 3 months (11). Pain intensity was quantified using the VAS, and the results were dichotomized for analysis.

For the purpose of statistical analysis, the pain outcome was dichotomized into “present” or “absent”. A case was defined as CPSP (positive) only if it met both of the following criteria: (1) VAS score ≥ 3; and (2) the pain was considered surgery-related. Pain with a persistent VAS score < 3 at 3 months, or pain with a VAS score ≥ 3 but assessed as unrelated to the surgery (e.g., new-onset arthritis, myofascial pain), was classified as non-CPSP. Furthermore, the regular use of analgesic medications (e.g., NSAIDs, weak opioids) for surgical site pain at 3 months was recorded and used to aid the clinical judgment of the pain's clinical significance and its relation to surgery.

(4) Other relevant variables

The occurrence of postoperative complications and the duration of chest tube placement were recorded.

Postoperative complications were assessed using the Clavien-Dindo classification system and were categorized into two groups, with Grade IIIa and above classified as one category. CPSP was defined according to the International Classification of Diseases, 11th Revision (ICD-11), as pain persisting for at least 3 months in the surgical area. The primary pain locations included tissues susceptible to traction and injury during surgery, such as the incision site, chest wall, and intercostal regions.

### 2.5 Statistical analysis

All statistical analyses were conducted utilizing SPSS Statistics version 26.0. Patient demographics, clinical characteristics, and surgical data were summarized descriptively: continuous measures were expressed as mean ± standard deviation, while categorical measures were reported as counts and percentages. Univariate analyses were first employed to assess the associations between various factors and postoperative chronic pain. Specifically, independent samples t-tests were used for continuous variables, and chi-square (*χ*^2^) tests were applied for categorical variables. All variables yielding a significance level of *P* < 0.05 in these univariate analyses were then entered into a multivariate logistic regression model to determine their independent effect on postoperative chronic pain, which was designated as the binary dependent variable. In this study, for pain score variables derived from multiple postoperative assessments (e.g., shoulder abduction pain), the maximum value recorded during the assessment period was used for modeling. Results from the logistic regression were reported as odds ratios (ORs) with corresponding 95% confidence intervals (CIs). For all analyses, a *P*-value of less than 0.05 was deemed to indicate statistical significance.

## 3 Results

### 3.1 Participant characteristics and group allocation

A total of 280 patients who underwent minimally invasive thoracic surgery were included in this study. Based on the presence of chronic postsurgical pain (CPSP) at the 3-month follow-up, they were categorized into two groups: the CPSP group (*n* = 48) and the non-CPSP group (*n* = 232). All enrolled patients completed the follow-up and were included in the final data analysis, with no loss to follow-up or missing data.

### 3.2 Study flow

**Figure 1:**
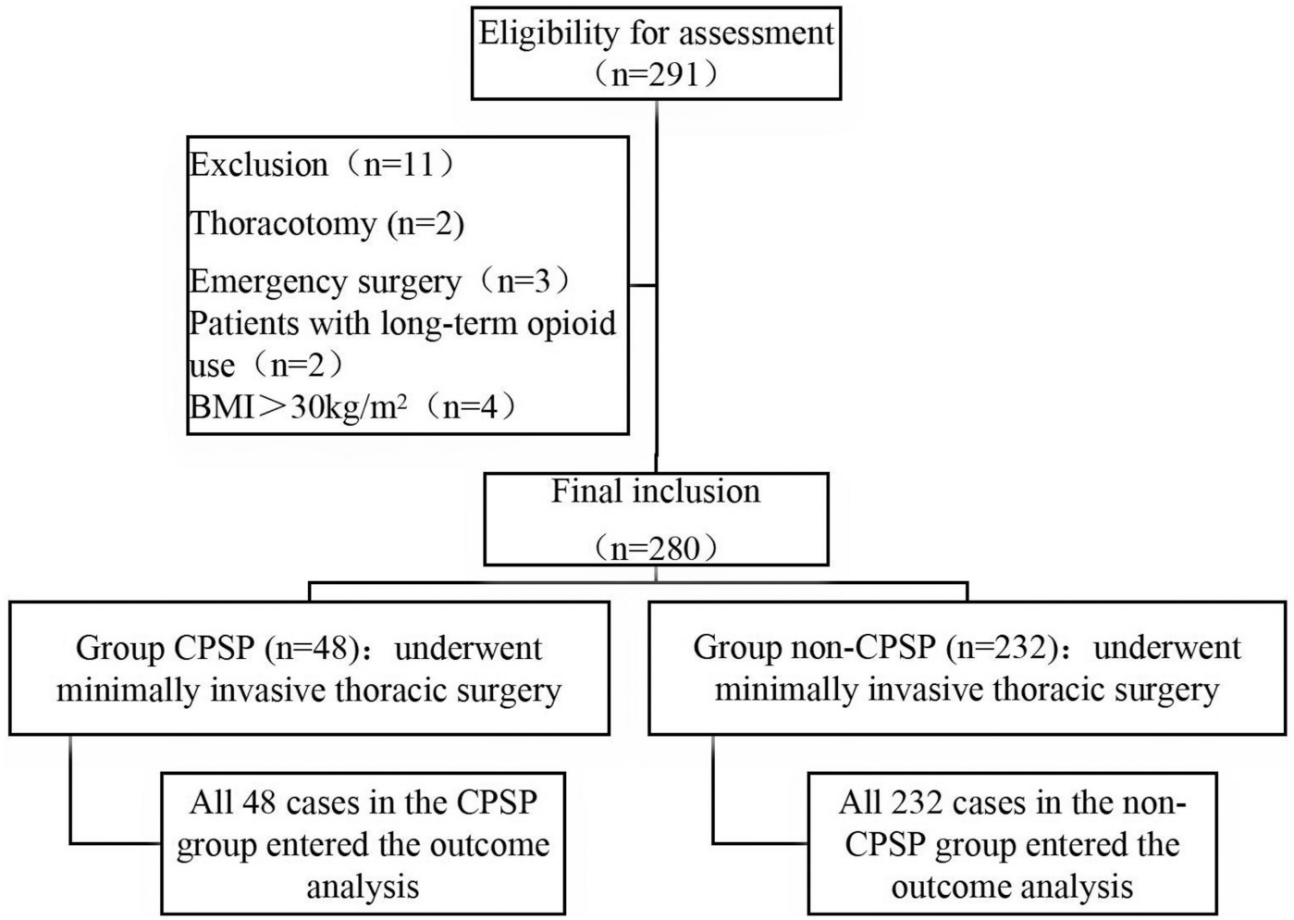
Flow chart of patient assignment.

The flow of participants through the study, from initial assessment to final group allocation, is detailed in Figure 1.

### 3.3 Univariate analysis of factors influencing CPSP

Univariate analysis revealed that the CPSP group and the non-CPSP group showed statistically significant differences (*P* < 0.05) with respect to preoperative anxiety, preoperative pain, use of intercostal suture, scar length, and postoperative shoulder abduction pain. These variables were consequently included in the subsequent multivariate analysis for further validation (see Table 1 for details).

**Table 1:** Univariate analysis comparing baseline characteristics, surgical features, and perioperative pain indicators between the two groups.

Indicator	CPSP group (*n* = 48)	Non-CPSP group (*n* = 232)	*χ*^2^/t	*P*
Baseline characteristics				
Male/female [*n*]	22/26	116/116	χ^2^ = 0.291	0.591
Age (years)	63.52 ± 5.24	62.83 ± 4.92	t = 0.892	0.375
BMI (kg/m^2^)	24.83 ± 2.53	25.12 ± 2.31	t = −0.754	0.454
ASA grade III [*n* (%)]	11 (22.9)	39 (16.8)	χ^2^ = 1.128	0.288
Preoperative anxiety [*n* (%)]	42 (87.5)	165 (71.1)	χ^2^ = 6.128	**0**.**013**
Surgical related indicators				
Surgical procedure [*n* (%)]			χ^2^ = 0.642	0.725
Lobectomy	30 (62.5)	152 (65.5)		
Wedge resection	12 (25.0)	58 (25.0)		
Segmentectomy	6 (12.5)	22 (9.5)		
Surgical approach [*n* (%)]			χ^2^ = 4.852	**0**.**028**
Axillary	32 (66.7)	115 (49.6)		
Posterolateral	16 (33.3)	117 (51.4)		
Duration of surgery (min)	168.52 ± 35.24	162.33 ± 32.73	t = 1.184	0.239
Intraoperative blood loss (mL)	152.36 ± 45.28	142.83 ± 42.16	t = 1.328	0.187
Intercostal suture [*n* (%)]	8 (16.7)	105 (45.3)	χ^2^ = 13.562	**<0**.**001**
Latissimus Dorsi preservation [*n* (%)]	18 (37.5)	105 (45.3)	χ^2^ = 0.942	0.332
Scar length (cm)	15.28 ± 3.45	12.36 ± 2.78	t = 5.842	**<0**.**001**
Surgical indication [*n* (%)]			χ^2^ = 2.187	0.535
Primary lung cancer	38 (79.2)	190 (81.9)		
Benign tumor	6 (12.5)	28 (12.1)		
Inflammatory/infectious	3 (6.3)	10 (4.3)		
Other	1 (2.1)	4 (1.7)		
Perioperative pain indicators				
Preoperative pain [*n* (%)]	30 (62.5)	85 (36.6)	χ^2^ = 11.284	**<0**.**001**
Postoperative pain trajectory [*n* (%)]			χ^2^ = 4.283	0.232
No change	16 (33.3)	62 (26.7)		
Rapid improvement	12 (25.0)	78 (33.6)		
Slow improvement	10 (20.8)	52 (22.4)		
Sharp deterioration	10 (20.8)	40 (17.2)		
Postoperative resting pain (points)	2.52 ± 0.83	2.31 ± 0.72	t = 1.782	0.077
Postoperative coughing pain (points)	2.12 ± 1.18	2.45 ± 1.24	t = −1.697	0.091
Postoperative shoulder abduction pain (points)	4.85 ± 1.36	2.64 ± 1.28	t = 9.842	**<0**.**001**
Postoperative complications				
Complications (≥grade IIIa) [*n* (%)]	3 (6.3)	8 (3.4)	χ^2^ = 0.832	0.362

Measurement data conforming to a normal distribution are presented as mean ± standard deviation and were compared using the independent samples *t*-test. Categorical data are presented as counts (percentages) and were compared using the χ^2^ test. *P* < 0.05 was considered statistically significant. indicates a statistically significant difference (*P* < 0.05). ASA, American Society of Anesthesiologists; BMI, body mass index.

Bold indicates the difference between the two groups is statistically significant.

### 3.4 Multivariate logistic regression analysis of factors influencing CPSP

The occurrence of chronic postsurgical pain (CPSP) was designated as the dependent variable. All six variables identified as significant in the univariate analysis were entered into a multivariate logistic regression model. The analysis ultimately identified postoperative shoulder abduction pain [OR = 1.893, 95% CI (1.432–2.502), *P* < 0.001], scar length [OR = 1.240, 95% CI (1.049–1.466), *P* = 0.011], and preoperative anxiety [OR = 3.089, 95% CI (1.201–7.943), *P* = 0.019] as independent risk factors for CPSP. In contrast, the use of the intercostal suture technique [OR = 0.234, 95% CI (0.074–0.736), *P* = 0.013] was identified as an independent protective factor. The results are detailed in Table 2.

**Table 2:** Results of the multivariate logistic regression analysis for chronic pain after minimally invasive thoracic surgery.

Variables	β	S.E	Z	*P*	OR (95%CI)
Intercept	−5.241	1.328	−3.945	<0.001	–
Postoperative shoulder abduction pain (points)	0.638	0.142	4.493	**<0.001**	1.893 (1.432–2.502)
Intercostal suture [yes]	−1.452	0.584	−2.486	**0.013**	0.234 (0.074–0.736)
Scar length (cm)	0.215	0.085	2.529	**0.011**	1.240 (1.049–1.466)
Preoperative anxiety [yes]	1.128	0.482	2.340	**0.019**	3.089 (1.201–7.943)
Surgical approach [axillary]	0.642	0.458	1.401	0.161	1.900 (0.774–4.664)
Preoperative pain [yes]	0.584	0.421	1.387	0.165	1.793 (0.786–4.091)

• Postoperative shoulder abduction pain was the strongest risk factor, with each 1-point increase in the VAS score associated with an 89.3% elevation in the risk of CPSP.

• The use of the intercostal suture technique was a protective factor, reducing the risk of CPSP by 76.6%.

• Each 1-cm increase in scar length was associated with a 24.0% increase in the risk of CPSP.

• The presence of preoperative anxiety was associated with a 2.1-fold increase in the risk of CPSP.

Bold indicates the difference between the two groups is statistically significant.

Model fitness tests yielded the following results: The likelihood ratio test (*χ*^2^ = 72.856, *P* < 0.001) indicated that the model containing the six independent variables had significant predictive value. The Hosmer-Lemeshow test (*χ*^2^ = 7.324, *P* = 0.501) suggested a good fit between the model's predicted probabilities and the observed outcomes. The overall prediction accuracy of the model was 84.3%. The Nagelkerke R^2^ value was 0.402, indicating that the model explained approximately 40.2% of the variance in CPSP occurrence. The Akaike Information Criterion (AIC) value was 198.45, demonstrating that the model maintained a concise variable structure while ensuring predictive capability.

### 3.5 Sensitivity analysis of predictive factors

Sensitivity analysis performed on the four independent influencing factors revealed that postoperative shoulder abduction pain possessed the best predictive performance [Area Under the Curve (AUC) = 0.821], with an optimal cut-off value of >3.5 points. At this threshold, it demonstrated high sensitivity (0.792) and specificity (0.784). Scar length also exhibited good predictive ability (AUC = 0.763), with an optimal cut-off value of >13.8 cm. Although preoperative anxiety had a relatively lower AUC (0.692), it demonstrated high sensitivity (0.875), making it suitable for use as a clinical screening indicator. The intercostal suture technique, as a protective factor, showed relatively limited predictive performance (AUC = 0.654); however, the absence of this technique demonstrated a good ability to identify potential CPSP cases. Model stability assessment, validated via Bootstrap resampling (1,000 iterations), showed that the 95% confidence intervals for the AUC of all variables did not include 0.5. The cross-validation accuracy was 82.7%, which was close to the original model's prediction accuracy of 84.3%, indicating that the model possesses good generalizability and stability. This analysis confirms that the four independent influencing factors have good robustness and clinical application value in predicting CPSP see Table 3.

**Table 3:** Predictive performance of independent influencing factors for CPSP.

Variables	AUC	Sensitivity	Specificity	Youden index	Optimal threshold
Postoperative shoulder abduction pain	0.821	0.792	0.784	0.576	>3.5 points
Scar length	0.763	0.708	0.724	0.432	>13.8 cm
Preoperative anxiety	0.692	0.875	0.509	0.384	Presence of anxiety
Intercostal suture	0.654	0.833	0.547	0.380	Not performed

## 4 Discussion

This case-control study systematically identified independent risk factors for Chronic Postsurgical Pain (CPSP) following minimally invasive thoracic surgery. Our multivariate logistic regression analysis demonstrated that postoperative shoulder abduction pain, scar length, and preoperative anxiety are significant independent risk factors, whereas the intercostal suture technique serves as a strong protective factor. These findings provide a quantitative basis for early identification of high-risk patients and targeted preventive strategies.

Our results demonstrate that, among the perioperative acute pain-related indicators, postoperative shoulder abduction pain exhibited the strongest predictive value (OR = 1.893, AUC = 0.821). This finding aligns with the conclusions of Rosenberger et al. (17), who suggested that perioperative acute pain indicators are strongly associated with CPSP. It is particularly noteworthy that in our study, the risk of CPSP increased significantly when the postoperative shoulder abduction pain score exceeded 3.5 points. This is comparable to the findings of Meredja et al. (18) in breast cancer surgery, whose team found that the maximum pain intensity on postoperative day 7 was more strongly correlated with CPSP than the pain intensity on postoperative day 1. Our study extends this conclusion to the field of thoracic surgery and further clarifies that activity-specific pain (shoulder abduction pain) has superior predictive value compared to the traditional assessment of pain at rest.

Postoperative shoulder abduction pain as a predictor for CPSP embodies three important attributes (1, 19). Regarding the timing of measurement, this study assessed it during the first 6 postoperative days. In terms of measuring intensity, quantification using the Visual Analog Scale (VAS) score facilitates clinical application and promotion (20). Regarding the pain type, pain during activity likely reflects the true extent of tissue damage more accurately than pain at rest (21). Under the common postoperative use of analgesic medications, activity-induced pain may better indicate the underlying pain-inducing conditions, such as the degree of tissue damage (22).

Regarding surgical technique, the intercostal suture technique demonstrated a significant protective effect (OR = 0.234). The study by Leandro et al. (23) showed that the intercostal suture technique significantly alleviated postoperative pain compared to traditional suturing methods. Our findings further substantiate the long-term preventive value of this technique against CPSP. Additionally, scar length, an objective indicator of surgical trauma, was identified as an independent risk factor (OR = 1.240). This positive correlation is supported by the work of Miyazaki (15) and other scholars. A longer incision typically implies more extensive tissue dissection and increased traction or injury to the intercostal nerves, consequently leading to a higher risk of neuropathic pain (24–27).

In terms of psychological factors, preoperative anxiety was confirmed as an important predictor for CPSP (OR = 3.089). Rosenberger et al. (17) pointed out that psychological factors play a key role in the development of CPSP. The single-question screening method used in this study, while simple, demonstrated high sensitivity, making it suitable for clinical practice. The mechanism by which anxiety promotes pain chronicity may involve multiple pathways. Dysfunction of the hypothalamic-pituitary-adrenal axis in anxious patients may enhance the sensitivity of peripheral nociceptors, while anxiety is often accompanied by impaired function of the descending pain inhibitory pathways, leading to reduced efficacy of the endogenous analgesic system (28, 29).

An important mechanism for CPSP development involves the transition from prolonged acute postoperative pain to chronic pain, potentially through mechanisms such as microglial polarization, ultimately leading to peripheral sensitization and corresponding central sensitization to noxious stimuli, as well as abnormal perception of non-noxious stimuli (30, 31). In support of this hypothesis, analgesic drugs used during general anesthesia or regional nerve blocks have been shown to influence the development of CPSP (32). Regarding analgesic drugs, intraoperative use of propofol is believed to reduce the incidence of CPSP. Lidocaine, ketamine, and gabapentinoids have also been reported to alleviate CPSP (10). Regarding regional blocks, techniques in various surgeries have proven effective in preventing CPSP development, although the mechanisms are not fully understood; they may reduce central sensitization and pain chronicity by modulating pain signals and neuroplasticity (33, 34).

In this study, all patients received standardized preoperative regional nerve blocks (TPVB/SAPB), reflecting current best-practice analgesic protocols within enhanced recovery pathways for thoracic surgery. Our data show no significant difference in the type of regional anesthesia technique between the CPSP and non-CPSP groups. This suggests that the independent risk factors (e.g., shoulder abduction pain, anxiety) and protective factors (intercostal suture) identified herein emerged against a background of high-quality, standardized foundational analgesia, enhancing the specificity and clinical relevance of these findings. Future research could further investigate the independent impact of different regional techniques or block quality itself on CPSP risk.Furthermore, all patients in this study followed a uniform, standardized postoperative multimodal analgesia protocol, and key metrics (e.g., postoperative opioid consumption) showed no significant difference between groups (Table 1). This further indicates that the strong association observed between early acute pain (e.g., shoulder abduction pain) and CPSP was not driven by inadequate or heterogeneous postoperative analgesia, but may reflect more profound tissue injury or neuro-sensitization processes. The standardized and adequate postoperative analgesic background enhances the independence and specificity of acute pain scores as predictors of CPSP in this study.

This study documented and analyzed the surgical indication (underlying diagnosis) as a baseline variable. Univariate analysis showed no significant difference in the distribution of major diagnostic categories (e.g., malignant vs. benign disease) between the CPSP and non-CPSP groups (*P* > 0.05, Table 1). Nevertheless, we acknowledge that a cancer diagnosis *per se* may act as a chronic psychological stressor, interacting with state anxiety to jointly influence pain perception and chronicity. Future research employing more nuanced tools (e.g., the Illness Perception Questionnaire) to disentangle diagnosis-related distress from trait/state anxiety could help clarify their relative contributions. The single anxiety-screening question used in this study may have captured elements of psychological distress related to a serious diagnosis.

Compared to previous studies, the innovation of this research lies in the simultaneous evaluation of the predictive value of multiple perioperative acute pain indicators for CPSP. However, the study has several limitations. The data source is limited to patients from a single center undergoing minimally invasive thoracic surgery. As the nature of acute and chronic pain—including intensity, incidence, and etiology—differs significantly across different types of surgery, the conclusions of this study cannot be readily generalized to other surgical procedures. Furthermore, the analysis of interactions between indicators was not sufficiently deep, partly due to the weak interaction effects observed and the current lack of standardized analytical procedures for such analyses. Future research should build upon this foundation by further expanding the sample size, including patients undergoing more types of surgery, and establishing more precise predictive models. Fourth, , although we analyzed and found the distribution of the primary surgeon to be balanced across pain outcome groups, and all surgeons followed the same technical principles, unmeasured variations in individual surgical execution details (e.g., suture tightness, tissue handling nuances) might remain as a potential residual confounding factor. Future multi-center studies could further control for surgeon-related variables.fifth, preoperative anxiety in this study was assessed using a single yes/no question rather than a validated psychological instrument such as the Hospital Anxiety and Depression Scale or the State-Trait Anxiety Inventory. While this method is simple and efficient for clinical practice and demonstrated high sensitivity in our study, it cannot quantify the severity of anxiety and may miss subthreshold symptoms. The use of validated scales might allow for more precise quantification of anxiety levels and potentially reveal a dose-response relationship between anxiety severity and CPSP risk, possibly yielding a stronger association. Nevertheless, the identification of anxiety as a significant risk factor even with this simple method suggests that anxiety detected through routine clinical screening warrants attention in perioperative pain management. Sixth, although all patients in this study followed a unified, opioid-sparing intraoperative anesthesia and analgesia protocol, and the consumption of key analgesics (e.g., opioids, NSAIDs) showed no statistical difference between the CPSP and non-CPSP groups, individual responses to medications and subtle pharmacodynamic variations might not be fully captured, representing a potential residual confounding factor. Future studies could consider more sophisticated pharmacokinetic/pharmacodynamic monitoring. Seventh, this study has limitations inherent to its retrospective design. The completeness and consistency of the routine clinical data relied upon may be inferior to that of a prospective design. Furthermore, several important variables known to potentially influence pain perception (e.g., genetic predisposition, psychosocial factors) were not systematically collected, which may lead to residual confounding. Therefore, the conclusions should be interpreted within this context. Future prospective studies should incorporate more comprehensive multidimensional data collection.

In conclusion, among various perioperative acute pain indicators, the shoulder abduction pain score measured over the first 6 postoperative days showed the strongest correlation with CPSP development and is the best candidate indicator for predicting CPSP. A postoperative shoulder abduction pain score exceeding 3 points was associated with CPSP occurrence. Intercostal suture is an independent protective factor against CPSP, while preoperative anxiety and greater scar length are risk factors. These findings provide an important basis for the early identification of high-risk patients and the formulation of targeted preventive strategies in clinical practice. It is recommended to enhance the monitoring and assessment of postoperative shoulder abduction pain, actively adopt the intercostal suture technique, and pay attention to patients’ preoperative psychological state, thereby effectively reducing the risk of CPSP, improving surgical outcomes, and enhancing patients’ quality of life.

## Data Availability

The raw data supporting the conclusions of this article will be made available by the authors, without undue reservation.
