# Supplementary material for: Risk factors for chronic postsurgical pain following minimally invasive thoracic surgery
Source: Front Surg. 2026 Jan 12;12:1742042. doi: 10.3389/fsurg.2025.1742042 (PMC12805442; doi:10.3389/fsurg.2025.1742042)
Supplement: Supplementary file 1 [file Supplementaryfile1.docx]

**Anesthetic program**

Upon arrival in the operating room, intravenous access was established. Standard monitoring included heart rate (HR), electrocardiography (ECG), pulse oximetry (SpO₂), and bispectral index (BIS). A peripheral intravenous line was secured, and an arterial catheter was inserted under local anesthesia into the radial artery of the non-operative side for continuous invasive blood pressure monitoring.

Anesthesia was induced intravenously with etomidate (0.3 mg/kg), sufentanil (0.5 μg/kg), and cisatracurium (0.2 mg/kg). After loss of consciousness and confirmation of adequate muscle relaxation, a double-lumen endotracheal tube of appropriate size was inserted using a video laryngoscope with the patient in the supine position. Correct positioning of the bronchial lumen was confirmed using fiberoptic bronchoscopy, followed by initiation of two-lung ventilation. Ventilator settings were as follows: fresh gas flow 2 L/min, tidal volume (VT) 8 ml/kg, respiratory rate (RR) 11-13 breaths per minute, positive end-expiratory pressure (PEEP) 5 cmH₂O, fraction of inspired oxygen (FiO₂) 100%, and inspiratory-to-expiratory ratio (I:E) of 1:2. End-tidal carbon dioxide (PETCO₂) was maintained between 35 and 45 mmHg.

Anesthesia was maintained with continuous intravenous infusions of propofol (3-5 mg·kg⁻¹·h⁻¹) and remifentanil (0.2-0.4 μg·kg⁻¹·min⁻¹), supplemented with intermittent intravenous boluses of cisatracurium (0.04 mg/kg) to maintain a BIS value between 40 and 60. Concomitantly, hemodynamic parameters were managed as follows: atropine (0.5 mg) was administered for HR <50 beats per minute; esmolol (0.5 mg/kg) was given for HR >100 beats per minute; phenylephrine (40 μg) was administered for systolic blood pressure (SBP) <90 mmHg or a decrease exceeding 20% from baseline; and for SBP >180 mmHg or an increase exceeding 20% above baseline, anesthetic depth was re-evaluated and urapidil (10 mg) was administered if necessary, with repeat dosing as required.

After repositioning the patient from supine to lateral decubitus, two-lung ventilation was continued, and the position of the double-lumen tube was reconfirmed via fiberoptic bronchoscopy. For one-lung ventilation (OLV), initiated upon surgical commencement, the breathing circuit was disconnected for 2 minutes concurrently with pleural opening to allow collapse of the operative lung. Ventilation was then switched to the non-operative lung. OLV settings were: fresh gas flow 2 L/min, VT 6 ml/kg, RR 14-18 breaths per minute, adjusted to maintain PETCO₂ between 35 and 45 mmHg.

Upon completion of skin closure, the infusions of propofol and remifentanil were discontinued, and the patient was transferred to the post-anesthesia care unit (PACU). In the PACU, neuromuscular blockade was reversed with intravenous neostigmine (0.04 mg/kg) and atropine (0.02 mg/kg). The patient was encouraged to awaken, and the double-lumen tube was extubated once the patient was responsive to simple commands and demonstrated adequate spontaneous respiration. After meeting the standard discharge criteria from the PACU, the patient was escorted back to the ward by an anesthesiologist.
